# Spontaneous expulsion from rectum: a rare presentation of intestinal lipomas

**DOI:** 10.1186/1749-7922-6-19

**Published:** 2011-06-13

**Authors:** Vasileios K Kouritas, Ioannis Baloyiannis, Georgios Koukoulis, Ioannis Mamaloudis, Dimitris Zacharoulis, Matheos Efthimiou

**Affiliations:** 1 Department of Surgery, Larissa University Hospital, Larissa, Greece

## Abstract

Lipomas are rare, subserosal, usually solitary, pedunculated small lesions appearing mainly in the large intestine with a minimal malignancy potential. They usually run asymptomatic and become symptomatic when they become enlarged or complicated causing intestinal obstruction, perforation, intusucception or massive bleeding. In rare cases they can be self-detached and expulsed via the rectum as fleshy masses. This event mainly occurs in large, pendunculated lipomas which detach from their pedicle. The reason for this event remains in most of cases unclear although in some cases a predisposing factor does exist. Abdominal pain and obstructive ileus may be observed while in many cases bleeding occurs. The expulsed mass sets the diagnosis and in most of the cases all symptoms subside. Diagnosis is rarely established before surgery with the use of barium enema, computed tomography and colonoscopy which additionally provides measures of treatment and diagnosis. In atypical cases though, in cases where the malignancy can not be excluded or in complicated cases, surgery is recommended. Usually the resection of the affected intestinal part is adequate. If during surgery a lipoma is encountered simple lipomatectomy seems also to be adequate.

## Background

Intestinal lipomas were firstly described by Bauer in 1757 [1] with 275 cases reported in the literature till 2001 [2]. They comprise a 5% of all gastrointestinal tract tumors [3,4]. Lipomas are considered to be the second most frequent benign lesions of the intestine appearing relatively rarely in clinical practice after adenomatous polyps [3-5]. Their malignant potential is considered to be minimal [3,4].

They are non-epithelial, mostly solitary, sessile or pedunculated lesions originating from mature lipocyte cells [6]. They can also appear in multiple locations in a 10-20% of cases especially if the lipoma is located in the ceacum [7,8]. They usually are small lesions, with a diameter less than 2 cm, but can reach a diameter of 30 cm [9,10] with most lesions being 4 cm at the time of detection [11]. They grow in the submucosal plane although occasionally they may extend into the muscularis propria, whereas in a 10% of cases they are subserosal [12]. They are covered either by an atrophic mucosa with congestion and inflammatory foci or are ulcerated with erosion of the overlying mucosa at the dome of the lipoma [13].

Their site of appearance starts from the hypopharynx till the rectum, with the ascending colon and the distal ileum alongside with the ileo-cecal valve being the most common sites of appearance [3,4,6,14-16]. The transverse, descending, sigmoid colon and rectum are other sites in order of greater appearance [17].

Lipomas present mainly on the right side of the abdomen with females in their 5^th ^decade of age being favored [11,18]. In males, the left abdomen is more often manifested [19].

## Presentation

Lipomas are long standing and usually run asymptomatic and unnoticed whatsoever for many years [6]. They become symptomatic in less than 30% of cases [4-6] and this usually occurs when they increase more than 2 or 3 cm in diameter [7,11]. It is reported that a 75% of patients with intestinal lipomas larger than 4 cm had symptoms [20]. In another study, 46% of the patients were diagnosed to have a lipoma by accidental diagnosis [21].

Patients complain of symptoms which are usually vague; the most frequent symptom reported is a non-specific abdominal pain with crabby, colic or intermittent character without rebound tenderness. This pain is usually repeated before the patient asks for medical assistance [1,3,4,6,7]. Constipation, altered bowel habits and hemorrhage are symptoms also often reported [4-6].

There is also lack of signs and findings during clinical examination [4-6]. It is possible to palpate a mass but this usually occurs when the lipoma is manifested with intussucception [13].

However, in most of the cases the lipomas are complicated and therefore the presenting symptoms and clinical signs appear according to the presenting manifestation, with hemorrhage being the most common symptom encountered [12].

The size of the lipoma plays key role in bleeding appearance possibility with lesions greater than 4 cm in diameter being presented with bleeding in 10% of cases [12]. Bleeding mainly occurs because of ulceration of the mucosal surface which covers the lipoma lesion. The underlying mechanism of ulcer development and consequently bleeding was proposed by Ginzburg [13]: the tumor at a time point starts to serve as the head for intusucception. This becomes congested and subsequent ulceration appears. Next, the mucosa covering the lipoma becomes ulcerated and the tumor is protruded beyond the mucosal plane forming a coronal border. In addition, this mechanism involves the formation of intussusception which is fairly true as lipomas predispose to intussusception which may also cause bleeding [5,22]. Blood loss from the gastrointestinal track may present as occult or chronic hemorrhage that may eventually lead to anemia, an event that is normally associated with intestinal malignancies [23]. In rare cases massive frank rectal bleeding may occur [7,17]. It must be noted that in some cases the bleeding can not be explained [12].

Symptoms and signs of ileal obstruction are also quite often seen. This is the case in masses evolving in the terminal ileus or in "giant" masses that cause lumen obstruction and ileus [4-6,24]. Moreover, giant lipomas interfere with stool passage producing changed bowel habit with bouts of diarrhea and constipation [25].

## Spontaneous expulsion

In rare cases the lipoma may be detached from its base and expulsed from the rectum.

This rare manifestation was firstly described in 1940 by Backenstoe with 19 cases being reported in the literature since 1942 [13].

Spontaneous expulsion of a lipoma is described only in few cases in literature [1,13,18,25-30]. We could retrieve less than ten cases published in the literature as single case reports whereas in most cases the spontaneous expulsion is mentioned apropos during presentation of lipoma series.

Spontaneous expulsion is observed in cases of huge lipomas which are mainly pedunculated with a narrow pedicle [26]. For an unknown reason, the lipoma is self-detached from its pedicle and becomes moveable within the ileal lumen interfering with stool passage and causing obstructive ileus. Another possible mechanism of self amputation suggests that when the ulceration of the mucosa above the lipoma is as large as its greatest diameter, consequently the below lying mass is protruded and detached into the lumen [13]. Eventually, the detached lipoma passes into the ascending colon and reaches the rectum from which it is expulsed with the feaces. There may also exists a reason for the amputation of the lipoma such as previous attempt of endoscopic removal [26] or intusucception [28,29] of the lipoma. As stated before in many cases, including our patient, the expulsion occurs for unknown reasons [13,24,27,30].

**Figure 1:**
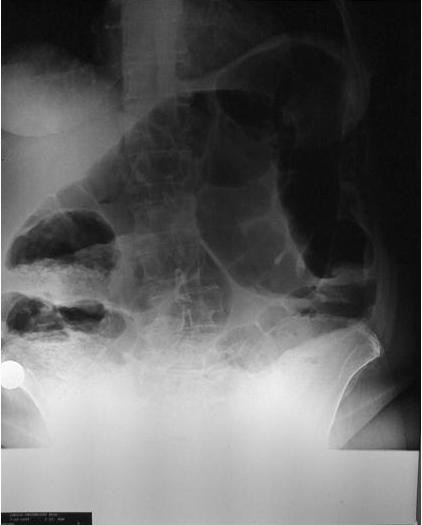
**Erect abdominal X-Ray of the patient at presentation**.

**Figure 2:**
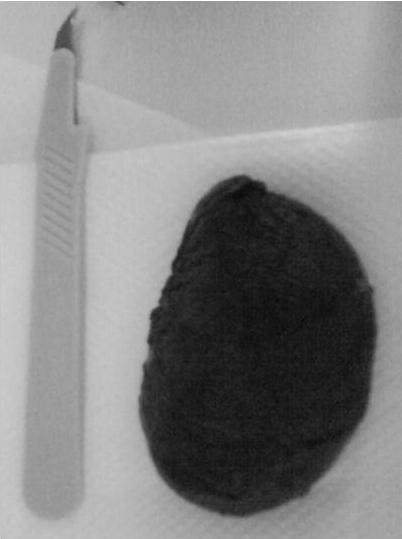
**The defecated mass a few hours after patient's presentation**.

The authors have also encountered one such case in a 77-year-old female who was presented with acute abdomen and melena (Figure 1) and who eventually expulsed a fleshy mass with her stool a few hours after initiation of the pain (Figure 2). Eventually her pain subsided after the expulsion and a thorough preoperative investigation was conducted including colonoscopy and barium studies.

This course of symptoms progression is more or less identical in most cases of spontaneous lipoma expulsion. The main symptom in most of the cases is abdominal pain usually left sided and colicky in character, followed by rectal bleeding [13,24,27-30] that subsides after defecation of the mass. In our case, the patient was presented with acute abdomen and melena.

Another possible presentation is obstructive ileus because the detached lipoma obstructs the ileo-ceacal junction and hinders stool passage [24]. In our case, the patient complained of constipation and inability to pass gasses and stool. On examination, his abdomen was distended with decreased bowel sounds.

Eventually, in almost all cases a fleshy mass is passed from the rectum and sets the diagnosis [24,27-30] as was the case in our patient.

In almost all cases the diagnosis was accidental either after examination of the expulsed mass [13,27,28,30] or during exploratory laparotomy [24,29]. In our case, the patient despite the expulsed tumor underwent laparotomy and right hemicolectomy because of the presence of multiple ulcers and lipomas observed in the ascending colon at colonoscopy which followed the mass expulsion.

## Diagnosis

Diagnosis of intestinal lipoma, if not accidental, is usually established during surgery for possible intestinal cancer or for treatment of lipoma complications [25,26].

In barium enema, an ovoid, well delineated, smooth and radiolucent mass is usually observed. The size and the shape of the mass may be changed with bowel movements with the elongation of the mass being the foremost appearance ("squeeze sign") [8]. In most cases, typical signs of intramular, extramucosal tumors are usually observed with a markely greater radiolucency because of the adipose tissue presence [13]. Diagnosis is achieved in less than 20% of cases [7].

Computed tomography will also show a spherical, ovoid, pear shaped mass with sharp margins with density of -40 to -120 Housfield units in uncomplicated cases [7,25]. In cases however with intusucception atypical imaging appearance may be encountered [31].

In colonoscopy, a normal lipoma may be visualized and therefore establish the diagnosis [26]. In more atypical cases, different observations may cause suspicion of the diagnosis [31]; the elevation of the mucosa over the mass with forceps ("tent sign"), the indentation of the lipoma with forceps ("cushion sign") or fat extrusion after biopsy ("naked fat sign"). Colonoscopy apart from diagnosis can provide a treatment modality especially in small lipomas less than 2 cm in diameter [6,7,25,26]. However, different approaches concerning the removal of the lipoma involve either the use of diathermia by which the stalk vessels can be thrombosed [26] or use of clips or loops [25,26]. The fact that fat is an inefficient electric current conductor and consequently hemorrhage may evolve should always be considered [7]. Additionally, the possibility of perforation seems to rise during colonoscopy and again should be considered [26]. Nevertheless, some authors believe that diagnosis is not eventually established because since lipomas are submucosal the biopsy performed will not involve tissue originating from deeper tissues [7].

MRI may provide additionally information but is not yet considered as a potential diagnosis indicator [7,25,26].

Despite all imaging modalities preoperative diagnosis is established in 62% of patients [32].

## Histopathology

In histopathology, mature and adult fat cells with lipoblasts surrounded by a fibrous capsule are usually observed [7]. "Pseudo-malignant" features may also be observed without however sarcomatous changes which are due to intermittent torsion and ischemia of the lesion [26].

## Treatment

The treatment modality is usually chosen according to the size of the mass, the complications or the suspicion of malignancy.

If the lipoma is less than 2 cm in diameter, it can be endoscopically removed, as stated before. For larger lesions more factors may play role apart from the size in choosing the correct modality such as the presence of a stalk (pedunculated lesions are easier removed than sessile lesions), the suspicion of malignancy or the manifestation of symptoms such as hemorrhage or obstruction [1,3,6,7,25,26]. The aforementioned factors if present consist endoscopic removal hazardous and therefore surgery should be preferred.

Surgery includes removal of the colon which is affected or more radical procedures such as hemicolectomy [6,33-36]. However, it should be noted that upon suspicion of a lipoma colotomy and lipomatectomy should be initially attempted [13]. Unfortunately, the lack of firm diagnosis before surgery and histopathology report leads to unnecessary laparotomies and colectomies [13].

Laparoscopic excision has been proposed to provide less postoperative pain, shorter duration of ileus and quicker recovery. Laparoscopic assisted minimally invasive techniques are also been reported in the treatment of lipomas [26,34,35].

Recurrence has not been so far documented [24].

## Conclusion

Intestinal lipomas are rarely appearing with their diagnosis being established postoperatively despite the imaging modalities available today. Although for small pendunculated lesions endoscopic removal seems adequate in most cases surgery is required to achieve excision, ensure diagnosis or to control manifestations such as obstruction or bleeding. Pedunculated lipomas may rarely detach from their base spontaneously and expulsed via the rectum, an event which although rare should not lead to cessation of further investigations.

## Consent

Written informed consent was obtained from the patient for publication of this case report and any accompanying images. A copy of the written consent is available for review by the Editor-in-Chief of this journal.

## Conflict of interests

The authors declare that they have no competing interests.

## Authors' contributions

IB, VKK, GK and ME treated and operated the patient. IB and VKK wrote the case report and the review. IM obtained the pictures. ME and DZ edited the paper. All authors have read and approved the final manuscript.
